# Relationship between health information literacy and health promoting lifestyle among first-degree relatives of patients with colorectal cancer in China: the mediating effect of health belief

**DOI:** 10.3389/fpubh.2023.1178848

**Published:** 2023-07-13

**Authors:** Jingru Zhou, Li Zhao, Yanjun Liu, Lin He, Fang Yang, Haichao Wang, Jing Fan, Qianer Li, Sisi Guo, Yanfen Wang, Yan Liu, Dan Zhou, Qin Tan

**Affiliations:** ^1^Department of Nursing, Deyang People's Hospital, Deyang, Sichuan, China; ^2^Department of Nursing, Affiliated Hospital of North Sichuan Medical College, Nanchong, Sichuan Province, China; ^3^Department of Infection, Mianzhu People’s Hospital, Mianzhu, Sichuan, China; ^4^Department of Nursing, Nanchong Health School, Nanchong, Sichuan, China; ^5^Department of Nursing, West China Hospital of Sichuan University, Chengdu, Sichuan, China

**Keywords:** colorectal cancer, first-degree relative, health belief, health-promotion lifestyle, health information literacy

## Abstract

**Background:**

History of first-degree relatives with colorectal cancer is one of the most important and common risk factors for colorectal cancer in China. Most chronic diseases, such as malignancies, are preventable by adopting health-promoting behaviors and other approaches. However, the relationships among factors affecting the health-promoting lifestyles of first-degree relatives with colorectal cancer have not been sufficiently studied. This study aimed to test the mediation effects of Health belief in the relationship between health-promoting lifestyle and health information literacy among first-degree relatives with colorectal cancer.

**Methods:**

A cross-sectional descriptive design was conducted using convenience sampling of 856 first-degree relatives of CRC patients attending three tertiary care hospitals in Nanchong and Deyang of China from December 2020 to December 2022. Questionnaires were used to collect data on the participants’ demographic information, the colorectal cancer health beliefs, the health promotion lifestyle, and the health information literacy. Data were analyzed with descriptive statistics, one-way ANOVA, Pearson’s correlation coefficients, and mediation analysis using SPSS 25.0 program and its macro-program PROCESS.

**Results:**

The findings indicated health information literacy was less, health belief was at the medium level, and performance of health promotion behavior was average for first-degree relatives of colorectal cancer. Whereas first-degree relatives of colorectal cancer health-promotion lifestyle had a positive correlation with health beliefs (*r* = 0.376, *p* < 0.01) and health information literacy (*r* = 0.533, *p* < 0.01), health beliefs had a positive correlation with health information literacy (*r* = 0.337, *p* < 0.01). Health beliefs mediated the positive effect of health information literacy on health-promoting lifestyles (β =0.420, 95% CI, 0.288–0.581), and indirect effects accounted for 14.0% of the total effect.

**Conclusion:**

Health information literacy and health beliefs are key factors associated with a health-promoting lifestyle among first-degree relatives with colorectal cancer. These factors have direct and indirect effects on each other and on health-promoting lifestyles. To enhance health-promoting lifestyles among first-degree relatives with colorectal cancer, interventions that strengthen health beliefs and provide health information literacy should be developed.

## 1. Introduction

The overall incidence of colorectal cancer (CRC) is increasing annually and it is now one of the most common cancers worldwide, making it a worldwide public health challenge. In 2020, there will be some 1.93 million new cases of CRC and approximately 930,000 deaths worldwide, ranking third in incidence and second in mortality among all malignancies (1). 2 million new CRC cases and 1.1 million deaths are expected worldwide by 2035 (2, 3). According to statistics published by the National Cancer Center in 2019, the incidence of CRC in China has shown a gradual increase over the past 30 years (4, 5), with about 388,000 new cases of CRC and 187,100 deaths in 2015, ranking third in cancer incidence and fifth in mortality in China. A history of first-degree relatives (FDRs) with CRC is one of the most important and common risk factors for CRC (6). It has been documented that first-degree relatives of CRC patients have a two to four fold increased risk of CRC compared to the general population (7). Approximately 25% of CRC cases occur in FDRs (8), and the higher the number of relatives with CRC, the higher the risk of FDRs (9).

The World Health Organization states that primary (e.g., sensible diet, physical activity) and secondary prevention strategies (e.g., screening, early detection) are effective means of reducing CRC incidence and mortality (10), thus showing that lifestyle behaviors and styles play an important role in human health (11, 12). Current research has also found that the development of CRC is closely related to an unhealthy lifestyle, such as irregular breakfast, low vegetable intake, poor diet such as consumption of red or processed meat, lack of exercise, overweight or obesity, smoking, and alcohol consumption (13, 14). Regular exercise can reduce the risk of CRC by 20%-30%, and proper nutrition (e.g., intake of a certain proportion of vegetables, fruits and cereals, dairy products, fish, etc.) can reduce the risk of CRC by 30%- 50% (15, 16). Therefore, one of the most effective means of preventing CRC is primary prevention, which is a health promotion lifestyle (10, 17, 18).

Health behaviors to prevent CRC vary across populations, but most of them are suboptimal. Koc et al. (9) showed that 51.7% of FDRs with CRC smoked, 31% of FDRs drank alcohol, only 44.7% of FDRs had a balanced diet, 20.2% of FDRs engaged in regular physical activity, and 16% of FDRs indicated that they were motivated to go to the hospital for routine check-ups. Jacobs et al. conducted a survey of 90 FDRs with CRC in the USA and found that 67% of FDRs reported that they adopted health promotion behaviors (19).

The Knowledge-Attitude-Belief-Practice (KABP) theoretical model is applied to the promotion of health behaviors (20). To change behavior, there must be knowledge (knowledge, information) as the basis and beliefs (correct beliefs, positive attitudes) as the motivation, through which people acquire relevant health knowledge and skills and gradually develop healthy beliefs and attitudes, which further contribute to the development of healthy behavior (21).

Foreign studies have shown that individuals who have more information about CRC risk factors and prevention methods have an increased commitment to adopt healthy behaviors to prevent CRC (22, 23). Less CRC health-related information is associated with lower levels of perceived CRC susceptibility and severity and negative health beliefs (24); individuals with higher health beliefs are more receptive to CRC prevention behaviors and are more likely to adopt healthy behaviors (25), which can promote proactive dietary and nutritional behaviors to prevent CRC (26).

Health information literacy emphasizes the ability to access, screen as well as use information as a tool to help individuals make better decisions. People’s lack of health information literacy can prevent them from accessing and understanding health information, leading to poor health beliefs. Lack of health information literacy has also been identified as a significant barrier to the adoption of health-promoting behaviors (27, 28). The World Health Organization says that information is the pathway to health and that health information literacy is a key element in promoting public health in the 21st century (29). The American Medical Library Association first introduced the concept of health information literacy (30). A survey by Hodges et al. (31) of people aged 50 years and older in the USA found that only 46.3% of the study participants had a high level of health information literacy. A cross-sectional survey study of US veterans by Omran et al. (24) showed that 36.3% of study participants with lower health information literacy had poorer CRC health beliefs, more negative attitudes towards CRC screening, and weaker health motivation, and that poor health information literacy is an important and often overlooked barrier to veterans taking up CRC screening. Pálsdóttir et al. (32) showed that health information literacy was positively associated with health promotion behavior in a study of 500 Icelanders, and research on health information literacy in China started relatively late. The level of health information literacy in different groups (e.g., older adult of patients with chronic diseases, breast cancer patients, and post-percutaneous coronary intervention patients) is low (33–35).

Health beliefs refer to a system of ideas that individuals hold about preventing disease, maintaining health, and striving for optimal living. The Health Belief Model (HBM) is a psychological model for explaining people’s health and illness-related beliefs and predicting health behaviors, which focuses on the role of perceptions (subjective judgments) in determining the formation and maintenance of health behaviors (36). It includes six aspects of perceived susceptibility, perceived severity, perceived benefits, perceived impairment, health motivation and self-efficacy (37, 38). Koc et al. showed that CRC patients with FDRs had higher levels of CRC health beliefs in Turkey (9). Jacobs et al. showed through a survey of 90 FDRs with CRC in the United States that most FDRs perceived CRC to be a serious disease, but they did not perceive themselves to be at risk of developing CRC, indicating a high level of perceived severity and a low level of perceived susceptibility (19). The findings of Bai et al. (39) and Leung et al. (40) showed that first-degree relatives of CRC patients and older residents had high levels of perceived benefits and self-efficacy for CRC screening in the community in Hong Kong, China. Xiaodan et al. (41) showed that the health beliefs of blood relatives with first-, second-and third-degree hereditary of CRC were at an intermediate level in Guangzhou, China.

Therefore, this study hypothesized that health beliefs plays a bridging role between health information literacy and health-promoting lifestyles, and applied the mediating effect model to explore the mediating role of health beliefs in the relationship between health information literacy and health-promoting lifestyles of first-degree relatives with CRC, and explored the relationship between the three, aiming to provide theoretical references for relevant departments or relevant personnel to improve health-promoting lifestyles of first-degree relatives with CRC.

## 2. Materials and methods

### 2.1. Design

This descriptive correlation study examined the effect of health information literacy on health-promoting lifestyles through the mediating effect of health beliefs in first-degree relatives of CRC.

### 2.2. Participants and procedure

First-degree relatives of CRC patients attending three tertiary care hospitals in Nanchong and Deyang were selected from December 2020 to December 2022. The inclusion criteria were as follows: (1) first-degree relatives of patients with CRC; (2) age ≥ 18 years; and (3) no cognitive impairment and normal expressive ability. The exclusion criteria were as follows: (1) those who had been diagnosed with malignancy; (2) first-degree relatives of patients with hereditary CRC; (3) those with serious organ damage, such as heart, kidney, and lung, in combination with mental disorders or abnormal behavior; and (4) those who were unwilling to participate in the survey. All participants provided written informed consent and a structured questionnaire with unified instructions was completed independently by the patients.

### 2.3. Sample size

According to the sample size calculation formula for the study of influencing factors of relevant variables is *N* = 4(*μ_α_s*/*δ*)^2^, where *μ_α_* is the *μ* value corresponding to the test level α, *S* is the standard deviation, and δ is the allowable error. Taking α = 0.05 and δ = 0.2S, the results of the pretest showed that the standard deviation S of the total score of the health-promoting lifestyle scale was 13.063. According to this calculation, *N* = 4*(1.96*13.063/2.612)^2^ = 385, considering 20% invalid questionnaires, the sample size was estimated to be at least 462. A total of 856 participants were included in this study.

### 2.4. Measures

#### 2.4.1. Demographic and medical characteristics

Designed by the researcher himself after reviewing relevant literature and consulting experts, the content included sex, age, education level, marital status, BMI, place of residence, occupation, *per capita* monthly income, whether there was a history of intestinal polyps, and whether he had received health education on colorectal cancer knowledge (whether participants attended health education seminars at the hospital and had registration information).

The diagnostically confirmed medical history of the participants was documented using a questionnaire including the history of intestinal polyps diseases. Detailed medical history (including history taking, review of previous colonoscopies, and medical records) was obtained. Participants who were unaware of their bowel condition underwent colonoscopies for diagnosis.

Height was measured using a TZG height gage with a precision of 0.1 centimeters (cm). Body weight was measured using an electronic calibrated scale (Tanita TBF-300A, Illinois, United States), accurate at 0.1 kg level. Participants were barefoot and dressed in light clothes. Body mass index, BMI is calculated by dividing weight (kg) by the square of height (m). Participants were classified into 4 categories according to the weight determination criteria for Chinese adults published by the National Health and Wellness Commission Participations, which were underweight (<18.5), healthy weight (18.5–24.9), overweight (25–27.9), and obese (≥28) (42).

#### 2.4.2. Health promoting lifestyle profile-II, revise, HPLP-IIR

This scale is a revised health-promoting lifestyle scale for the Chinese population obtained by Cao et al. (43), which was further modified from the HPLP-II. The scale was used to measure the level of health-promoting lifestyles of the study participants. The scale consists of 6 dimensions and 40 items, namely, interpersonal relationships (5 items), nutrition (6 items), health responsibility (11 items), physical activity (8 items), stress management (5 items), and spiritual growth (5 items). Each item is scored on a 4-point Likert scale. The score ranges from 40 to 160, with higher scores indicating higher health-promotion behavior. The total score was divided into 4 levels, with 40–69 being poor, 70–99 being fair, 100–129 being good, and 130–160 being excellent (44). The split-half reliability of each dimension was 0.640–0.780, the Cronbach’s *α* coefficient was 0.630–0.810, and the scale retest reliability was 0.690. Cronbach’s alpha coefficient for the scale measured in this study was 0.938.

#### 2.4.3. Colorectal Cancer health belief scale (CCHBS)

This scale was developed by Jacobs (19) on the basis of the Champion Health Beliefs Scale and was Chineseized and validated for reliability by Xiaodan (45). The scale consists of six dimensions with 36 entries: perceived susceptibility (5 entries), perceived severity (7 entries), perceived benefits (6 entries), perceived barriers (6 entries), health motivation (7 entries), and self-efficacy (5 entries). The Likert 5-point scale was used, with scores ranging from 1 to 5 on a scale of “completely disagree” to “completely agree,” with the perceived impairment dimension being scored in reverse. Higher scores indicated higher beliefs about the health of CRC. *Cronbach’s alpha* coefficient for this scale (Chinese version) was 0.881, and the content validity index (S-CVI) was 0.980 (45). In this study, internal consistency reliability was acceptable (α = 0.794).

#### 2.4.4. The Chinese version of the health information literacy self-rating scale (HILSS)

The scale was developed by the Chinese scholar Wang et al. (46), and includes a comprehensive consideration of the Chinese population in terms of information access and information behavior characteristics. It includes 29 items and five domains: health information consciousness (four items, HIC), health information access (twelve items, HIS), health information evaluation (five items, HIE), health information applications (four items, HIA), and health information morality (four items, HIM). The Likert scale was used, with entries assigned rating values quantified in the [0, 1] range for a total of five levels. The higher the total score, the higher the individual’s level of health information literacy of the individual. The results were processed on a percentage scale, with a score above 60 indicating a competent level of health information literacy (46). The *Cronbach’s* alpha value of the scale was 0.847. Cronbach’s alpha coefficient for the scale measured in this study was 0.869.

### 2.5. Procedure

The study was approved by the Ethics Committee of the Affiliated Hospital of North Sichuan Medical College. The researcher contacted the department director, head nurse, or doctor of the relevant departments to obtain their support. Following the principles of voluntary participation and informed consent, data were collected by face-to-face questionnaire for study subjects who met the inclusion criteria. The researcher himself introduces the purpose, significance, filling method, and time spent on the study, and invites the research object to sign the informed consent and issue the questionnaire. The researcher checks the questionnaire on the spot.

### 2.6. Statistical analysis

We used descriptive statistics to identify demographic and health beliefs, health-promoting lifestyles, and health information literacy, such as frequency, percentage, mean, and standard deviation. Second, one-way ANOVA was used to assess whether different categories were different for health beliefs, health-promoting lifestyles, and health information literacy. Furthermore, we studied the relationships between health beliefs, health-promoting lifestyles, and health information literacy using Pearson’s correlation. We used the SPSS PROCESS Macro Program to analyze health beliefs as a mediator of the effect of health information literacy on health promotion lifestyles, with health promotion lifestyles as the dependent variable (Y), health information literacy as the independent variable (X), and health beliefs as the mediating variable (M). Furthermore, Model 4 was selected, and bootstrapping was used to test the statistical significance of the coefficient; bias-corrected 95% confidence intervals (BC 95%CI) were applied to the values obtained from 5,000 bootstrap samples. The analysis facilitated the estimation of the indirect effect using a normal theory approach and a bootstrap approach to obtain confidence intervals (47). We further analyzed the data collected using SPSS25.0 and set the significance level at.05 for all the analyses.

## 3. Results

### 3.1. Demographic characteristics

The characteristics of the sample are listed in Table 1. In the 856 respondents in this study, the mean age of patients was 41.91 (SD =10.65) years. The 776 participants were children, 52 participants were close siblings, and 28 participants were parents of CRC patients. There were 492 males (57.4%) and 364 females (42.6%), and 756 married first-degree relatives (83.7%). The normal test results and scores of the variables are shown in Table 2. For each of the observed variables, the kurtosis and skewness values were between −1 and 1; therefore, the sample can be considered to have a normal distribution.

**Table 1:** The participants’ general demographic and characteristics (*N* = 856).

Variable	*n*	Percent	HPLP-II R			CCHBS			HILSS		
			Mean ± SD	t OR F	*p* Tukey	Mean ± SD	t OR F	*p* Tukey	Mean ± SD	t OR F	*p* Tukey
Intestinal polyps											
No	768	89.7	99.97 ± 16.36	4.353	<0.001	121.74 ± 10.39	1.382	0.170	17.13 ± 2.89	4.267	<0.001
Yes	88	10.3	91.86 ± 18.74			118.86 ± 19.58			15.72 ± 3.39		
Sex											
Male	492	57.4	97.92 ± 15.97	−2.474	0.014	119.86 ± 13.17	−4.953	<0.001	16.90 ± 2.84	−0.913	0.361
Female	364	42.6	100.78 ± 17.72			123.58 ± 8.80			17.09 ± 3.14		
Age (years)											
≤40	380	44.4	99.35 ± 17.33	0.338	0.736	121.12 ± 9.30	−0.753	0.452	17.44 ± 2.84	4.099	<0.001
>40	476	55.6	98.96 ± 16.35			121.70 ± 13.25			16.62 ± 3.03		
Marital status											
Unmarried	100	11.6	96.88 ± 16.71	−1.439	0.153	116.40 ± 9.89	−4.679	<0.001	17.30 ± 2.36	1.362	0.175
Married	756	88.4	99.43 ± 16.75			122.11 ± 11.67			16.94 ± 3.03		
Education											
Primary school and below	92	10.8	95.82 ± 17.08	14.095	<0.001	119.78 ± 17.25	1.335	0.262	15.41 ± 2.65	60.686	<0.001
Junior High School	300	35.0	97.06 ± 14.72			121.14 ± 11.37			16.19 ± 2.55		
High school	220	25.7	97.00 ± 16.58			121.41 ± 10.13			16.61 ± 2.73		
University and above	244	28.5	104.86 ± 17.71			122.47 ± 10.47			18.88 ± 2.85		
BMI(kg/m^2^) status											
Thin	48	5.6	104.16 ± 18.09	3.524	0.015	125.83 ± 8.73	5.028	0.002	18.31 ± 2.25	7.490	<0.001
Normal	508	59.3	98.54 ± 17.92			121.57 ± 11.18			16.89 ± 3.10		
Overweight	224	26.2	100.66 ± 13.89			121.50 ± 12.09			17.28 ± 2.73		
Obesity	76	8.9	95.47 ± 14.15			117.68 ± 13.55			15.92 ± 2.74		
Household income(monthly)											
<1,500 RMB	152	17.8	100.57 ± 20.39	5.682	<0.001	121.18 ± 16.31	3.174	0.024	15.79 ± 3.04	18.794	<0.001
1,500 ~ 3,000 RMB	256	29.9	97.35 ± 15.30			121.01 ± 11.64			16.91 ± 2.68		
>3,000 ~ 4,500 RMB	208	24.3	96.69 ± 16.13			123.51 ± 7.93			16.80 ± 3.21		
>4,500 RMB	240	28.0	102.25 ± 15.60			120.28 ± 10.50			17.99 ± 2.65		
Occupation											
Employees/Retirees of government and institutions	120	14.0	105.00 ± 19.25	10.396	<0.001	124.76 ± 11.00	5.902	0.003	18.79 ± 3.20	51.978	<0.001
Farmers	252	29.4	96.65 ± 16.92			120.58 ± 14.78			15.71 ± 2.64		
Other professionals	484	56.6	98.98 ± 15.60			121.07 ± 9.61			17.20 ± 2.78		
Religious beliefs											
Has	56	6.5	93.21 ± 21.77	−2.137	0.006	117.00 ± 16.67	−2.104	0.040	15.56 ± 2.77	−3.727	<0.001
No	800	93.5	99.55 ± 16.25			121.76 ± 11.13			17.08 ± 2.95		
Residence											
Rural	364	42.5	96.00 ± 16.80	−4.784	<0.001	120.40 ± 13.16	−2.181	0.030	16.22 ± 2.69	−6.718	<0.001
City	492	57.5	101.46 ± 16.30			122.21 ± 10.27			17.55 ± 3.04		
Commercial Insurance											
Yes	180	21.0	102.37 ± 16.53	2.935	0.003	122.95 ± 12.03	1.961	0.050	17.96 ± 2.51	5.620	<0.001
No	676	79.0	98.27 ± 16.68			121.04 ± 11.48			16.72 ± 3.03		
Relatives working in the medical field											
Yes	208	24.3	102.65 ± 17.01	3.504	<0.001	123.98 ± 10.44	3.683	<0.001	17.80 ± 3.20	4.594	<0.001
No	648	75.7	98.01 ± 16.49			120.63 ± 11.86			16.72 ± 2.84		
Health education about colorectal cancer											
Yes	108	12.6	108.22 ± 15.44	6.164	<0.001	126.66 ± 12.09	5.063	<0.001	19.28 ± 3.27	7.961	<0.001
No	748	87.4	97.82 ± 16.50			120.69 ± 11.36			16.65 ± 2.77		

**Table 2:** Descriptive statistics of HPLP-II R, CCHBS, and HILSS (*N* = 856).

Variable	Actual score range	Total scores	Mean item score
HPLP-II R	41 ~ 147	99.14 ± 16.75	2.47 ± 0.41
Interpersonal relationships	5 ~ 20	14.13 ± 2.47	2.82 ± 0.49
Health responsibility	11 ~ 40	24.05 ± 5.13	2.18 ± 0.47
Stress management	5 ~ 19	12.52 ± 2.47	2.50 ± 0.49
Nutrition	7 ~ 24	16.13 ± 3.06	2.68 ± 0.51
Physical activity	8 ~ 31	17.84 ± 4.57	2.23 ± 0.57
Spiritual growth	5 ~ 20	14.44 ± 2.72	2.88 ± 0.54
CCHBS	68 ~ 160	121.44 ± 11.64	3.37 ± 0.32
Perceived susceptibility	5 ~ 23	12.36 ± 3.52	2.47 ± 0.70
Perceived severity	7 ~ 33	21.50 ± 5.09	3.07 ± 0.72
Perceived benefits	6 ~ 30	23.57 ± 3.72	3.92 ± 0.62
Barriers	9 ~ 30	19.80 ± 4.34	3.30 ± 0.72
Health Motivation	7 ~ 35	26.57 ± 3.92	3.79 ± 0.56
Self-efficacy	5 ~ 25	17.62 ± 3.15	3.52 ± 0.63
HILSS	9.48 ~ 27.00	16.98 ± 2.97	0.58 ± 0.10
Health information consciousness	1.50 ~ 4.00	2.77 ± 0.49	0.69 ± 0.12
Health information access	1.65 ~ 11.60	6.26 ± 1.86	0.52 ± 0.15
Health information evaluation	1.50 ~ 4.60	3.10 ± 0.56	0.62 ± 0.11
Health information applications	1.08 ~ 4.00	2.55 ± 0.58	0.63 ± 0.14
Health information morality	1.00 ~ 3.25	2.29 ± 0.39	0.57 ± 0.10

The analysis of differences in health-promoting lifestyles, according to participants’ general characteristics, showed significant variations based on whether there was a history of intestinal polyps (*t* = 4.353, *p* < 0.01), sex (*t* = −2.474, *p* < 0.05), educational level (*F* = 14.095, *p* < 0.01), BMI (*F* = 3.524, *p* < 0.05), household income (monthly) (*F* = 5.682, *p* < 0.01), occupation (*F* = 10.396, *p* < 0.01) religious beliefs (*t* = −2.137, *p* < 0.01), residence (*t* = −4.784, *p* < 0.01), commercial Insurance (*t* = 2.935, *p* < 0.01), relatives working in the medical field(*t* = 3.504, *p* < 0.01), and health education about colorectal cancer (*t* = 6.164, *p* < 0.01).

The analysis of differences in health beliefs, according to participants’ general characteristics, showed significant variations based on sex (*t* = −4.953, *p* < 0.01), marital status (*t* = −4.679, *p* < 0.01), BMI (*F* = 5.028, *p* < 0.01), household income (monthly) (*F* = 5.682, *p* < 0.05), occupation (*F* = 5.902, *p* < 0.01), religious beliefs (*t* = −2.104, *p* < 0.05), residence (*t* = −2.181, *p* < 0.05), relatives working in the medical field (*t* = 3.683, *p* < 0.01), and health education about colorectal cancer (*t* = 5.063, *p* < 0.01).

The analysis of differences in health information literacy, according to participants’ general characteristics, showed significant variations based on whether there was a history of intestinal polyps (*t* = 4.267, *p* < 0.01), age (*t* = 4.099, *p* < 0.01), educational level (*F* = 60.686, *p* < 0.01), BMI (*F* = 7.490, *p* < 0.01), household income (monthly) (*F* = 18.794, *p* < 0.01), occupation (*F* = 51.978, *p* < 0.001), religious beliefs (*t* = −3.727, *p* < 0.01), residence (*t* = −6.718, *p* < 0.01), commercial insurance (*t* = 5.620, *p* < 0.05), relatives working in the medical field (*t* = 4.594, *p* < 0.01), and health education about colorectal cancer (*t* = 7.961, *p* < 0.01).

### 3.2. Correlations among health promotion lifestyle, health beliefs, and health information literacy

The mean health promotion lifestyle score of first-degree relatives was 99.14 (SD = 16.75), and the highest subdomain scores were for spiritual growth (2.88, SD = 0.54). The mean CCHBS score was 121.44 (SD =11.64). The mean HILSS score was 16.98 (SD = 2.97; Table 2).

Correlation analysis showed that health promotion lifestyle was positive associated with health beliefs (*r* = 0.376, *p* < 0.01) and health information literacy (*r* = 0.533, *p* < 0.01). Health information literacy had a significant positive association with health beliefs (*r* = 0.337, *p* < 0.01; Table 3).

**Table 3:** Correlations between health promotion lifestyle, health beliefs, and health information literacy (*r*, *N* = 856).

Variables	Health beliefs	HPLP-II R	Health information literacy
Health beliefs	1		
HPLP-II R	0.376^**^	1	
Health information literacy	0.337^**^	0.533^**^	1

^**^p < 0.01.

### 3.3. The mediating effect of health beliefs on the relationship between health information literacy and health promoting lifestyle among FDRs of patients with CRC

**Figure 1:**
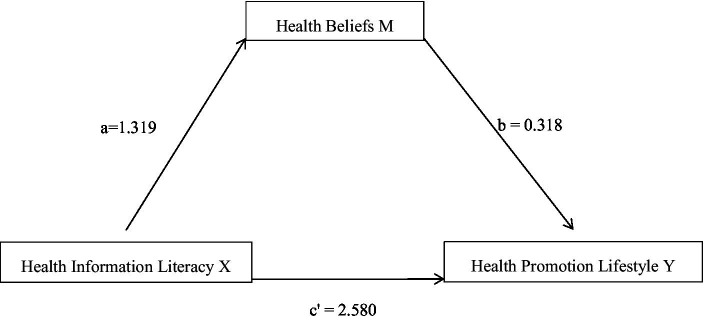
The mediating effect of health information literacy on health promotion lifestyle through health beliefs. There was a significant indirect effect of health information literacy on health beliefs through health beliefs, *β* = 0.420, 95% BCaCI [0.288–0.581].

Furthermore, Model 4 was selected, and bootstrapping was used to test the statistical significance of the coefficient; bias-corrected 95% confidence intervals (BC 95%CI) were applied to the values obtained from 5,000 bootstrap samples, and the parameters were set to obtain three regression path models, as shown in Table 4; Figure 1. The path coefficient from health information literacy (X) to health beliefs (M) was statistically significant (*β* = 1.319, *p < 0.01*), while the path coefficient from health information literacy (X) to health promoting lifestyles (Y) was statistically significant (*β* = 3.001, *p < 0.01*). The path coefficient from health information literacy (X) and health beliefs (M) to health promoting lifestyles (Y) was statistically significant (*β*_b_ = 0.318, *β*_c’_ = 2.580, *p < 0.01*), indicating a partial mediating effect of health beliefs (M; see Table 4). The total indirect effects were 3.001 [95%confidence interval (CI), 2.680-3.321]. Health beliefs (M) mediated the positive effect of health information literacy (X) on health-promoting lifestyles (Y) (*β* = 0.420; 95% CI, 0.288–0.581), and indirect effects accounted for 14.0% of the total effect.

**Table 4:** Mediating effects of health beliefs of first-degree relatives with colorectal cancer between health information literacy and health-promoting lifestyles pathway model analysis (*N* = 856).

Path model	Dependent variable	Independent variable	*R* ^2^	*p*-value	Partial regression coefficient	Standard error	*t*-value	*p*-value	95% CI
X-M	Health beliefs	Health Information Literacy	0.113	<0.001	1.319	0.126	10.460	<0.001	1.072 to 1.566
X-Y	Health Promoting Lifestyles	Health Information Literacy	0.283	<0.001	3.001	0.163	18.384	<0.001	2.680 to 3.321
X, M-Y	Health Promoting Lifestyles	Health Information Literacy	0.327	<0.001	2.580	0.168	15.347	<0.001	2.250 to 2.910
		Health beliefs			0.318	0.043	7.421	<0.001	0.234 to 0.403

## 4. Discussion

The results of the current study showed that the health promotion lifestyle score of first-degree relatives with CRC was 99.14 ± 16.75, which was the overall average for the study population according to the rating scale of the total scale score, and was consistent with the findings of the study by Junting et al. (48) investigating a high-risk population with gastric cancer and Lan et al. (49) investigating a high-risk population with stroke, and lower than the findings of the studies of Huimin et al. (50) and Bieyabanie et al. (51) on cancer patients. The reason for this may be that cancer patients understand the importance of health, pursue healthy behavior, and maintain good lifestyle habits after their illness. Relevant departments and personnel should promote the importance of a healthy lifestyle and provide adequate education on health-promoting lifestyles to first-degree relatives with CRC and should also assume appropriate monitoring responsibilities. Relevant departments and personnel can recommend aerobic exercises (such as walking, jogging, square dancing, and Tai Chi) that are easily acceptable and easy to adhere to, attach importance to training on stress management for people at high risk of CRC (such as training on stress reduction, psychological counselling, reasonable catharsis, use of techniques to relieve stress, and other methods), promote a reasonable diet, and guide first-degree relatives to establish health responsibilities; they can also further strengthen health education and promotion campaigns with innovative use of media, public campaign facilities, and public awareness programs (52) to improve the health-promoting lifestyle of first-degree relatives with CRC.

The results of this study showed that the health belief score of first-degree relatives with CRC was 121.44 ± 11.64, with a mean entry score of 3.37 ± 0.32, which was at an intermediate level, consistent with the findings of Xiaodan et al. (41) and higher than those of Li et al. (53) and Lin et al. (54), probably because the respondents of Li et al. (53) and Lin et al. (54) were ordinary community residents; in Taiwan, they had less contact with CRC patients and medical personnel and did not have the opportunity to actively or passively receive education on the knowledge; thus, the level of health beliefs about CRC was lower.

The results of this study showed that the health information literacy score of the first-degree relatives of CRC patients was 16.98 ± 2.97, and 500 peoples (58.4%) scored less than 60 after the results were processed on a percentage scale, indicating that 58.4% of the first-degree relatives of CRC patients lacked health information literacy (55), which shows that the health information literacy of this study population is lacking. The reason for this is that the majority of the population in this study were older adult, who are generally less educated and less able to learn and are influenced by traditional concepts and solidified thinking, which makes it more difficult for them to obtain, cognise, evaluate, and apply health information. The health information access dimension had the lowest score, with a mean score of 0.52 ± 0.15, which is at a low level, indicating that the population in this study had poor ability to assess the quality of health information and its usability in specific settings. The highest mean score for the health information perception dimension was 0.69 ± 0.12, which is at a medium level, indicating that the population in this study has an average ability to correctly understand health information needs, and only has a preliminary ability to identify health information sources and search for relevant information, and therefore needs further improvement.

The results of the correlation analysis in this study showed that health beliefs were positively correlated with health information literacy (*r = 0*.337, *p < 0.01*)*, health information literacy was positively* correlated with health promoting lifestyles (*r* = 0.533, *p* < 0.01), and health beliefs were positively correlated with health promoting lifestyles (*r* = 0.376, *p < 0.01*). Bootstrap mediation analysis further revealed that health information literacy of first-degree relatives with CRC was a positive predictor of health promotion lifestyle (*β = 2*.580, *p < 0.01*), and health information literacy could also indirectly affect health promotion lifestyle by influencing the health beliefs of first-degree relatives with CRC (indirect effect value of 0.420), accounting for 14.0% of the total effect of the total effect. People with low health information literacy have a single source of health information and have difficulty obtaining the information they want from cancer prevention information, materials, and conversations (27), whereas colorectal first-degree relatives with health information literacy are aware of the value of health information and actively seek out colorectal cancer-related information. Good health information search skills help people at risk to try to access colorectal cancer-related health information in multiple ways and in multiple ways to obtain health knowledge related to CRC, the better the ability to access information, the more health information they obtain, and the ability to discriminate between health information, which helps them to develop positive health beliefs and adopt healthy behaviors to maintain and promote health (56).

Health beliefs are key for people to accept persuasion, change undesirable behaviors, and adopt healthy behaviors (57). Effective communication of health information plays an important role in health promotion and cancer prevention, and it is necessary to explore specific programs in various aspects, such as methods of providing effective information and appropriate timing of provision, to help first-degree relatives with CRC have the right information and improve health beliefs and behaviors. The United States, Canada, and Italy have successively launched health information literacy education programs and set up hospital librarians for patients to help people with low health information literacy access effective health information (58, 59), and relevant departments and personnel can learn from such experiences and measures. In the future, it is recommended that relevant departments and personnel should pay more attention to first-degree relatives with CRC who lack health information literacy and can use convenient channels such as information technology to provide knowledge about CRC, specific skills for health behaviors, and also provide them with access to high-quality health information (e.g., public websites and health medicine websites). The government should also pay attention to the improvement of the health information literacy level of first-degree relatives with CRC, and provide educational interventions targeting their health information ethics, health information application, and health information evaluation skills. Government departments need to make efforts to conduct popular education on knowledge of Internet information retrieval and accelerate the construction of user-friendly medical Internet information retrieval devices, provide professional services and training skills to strengthen the awareness and access to health information of first-degree relatives with CRC, and the ability to access health information. Health education based on the health belief model can improve CRC health beliefs, promote screening behavior, and prevent CRC prevention behavior (16, 60). Health education based on the Health Belief Model should be provided by hospitals or community staff to first-degree relatives with CRC. It can take various forms, such as lectures, seminars, videos, and websites, to provide prevention knowledge, disease knowledge, and health promotion behavior methods, and promote mutual supervision among family members to promote good health beliefs among first-degree relatives with CRC, so as to effectively improve poor lifestyles and establish good health promotion behavior.

## 5. Limitations

Due to time and condition constraints, only first-degree relatives with CRC in three tertiary care hospitals in Nanchong and Deyang City were selected for this study, and social venue studies were not included; therefore, representation was limited. This was a cross-sectional study and did not provide a good understanding of the dynamic changes in health promotion lifestyle-related variables among first-degree relatives with CRC. Longitudinal studies can be conducted in the future to further clarify the dynamic changes in the mechanism of action of health promotion lifestyle-related variables among first-degree relatives with CRC and to expand the ideas and depth of the study.

## 6. Conclusion

(1) The health information literacy of first-degree relatives with CRC is low. Health beliefs of first-degree relatives are at an intermediate level. Health promotion behaviors of first-degree relatives are average. (2) The study results showed that when health information literacy are higher, health beliefs is higher, and so is health promotion lifestyles. (3) In addition, health information literacy and health beliefs affect health-promoting lifestyle, and health beliefs mediates the relationship between health information literacy and health-promoting lifestyle.

## Data availability statement

The raw data supporting the conclusions of this article will be made available by the authors, without undue reservation.

## Ethics statement

The studies involving human participants were reviewed and approved by the Ethics Committee of the Affiliated Hospital of North Sichuan Medical College approved the study (approval number: 2020ER186-1). The patients/participants provided their written informed consent to participate in this study. Written informed consent was obtained from the individual(s) for the publication of any potentially identifiable images or data included in this article.

## Author contributions

JZ and LZ designed the study, analyzed and interpreted the data, and drafted the manuscript. JZ, YjL, SG, and DZ formally analyzed and investigated. JZ, YjL, and JF worked on study design and data collection and helped with manuscript preparation. HW, LZ, QT, and FY lent substantial support in the analysis and interpretation of the data. QL, JF, FY, YW, LH, and YaL contributed to the critical revision of the report. LH final approval of the version to be published. All authors contributed to the article and approved the submitted version.

## Funding

Funding for this study was provided by Sichuan Mental Health Education Research Center Research Project (XLJKJY2049C), Nanchong City Social Science Research “14th Five-Year Plan” 2022 annual project (NC22B298), Scientific Research Development Program of Affiliated Hospital of North Sichuan Medical University (2021SK002), Natural Science Project of Sichuan Nursing Vocational College (2022RZY40). Funding body played a role in the design of the study.

## Conflict of interest

The authors declare that the research was conducted in the absence of any commercial or financial relationships that could be construed as a potential conflict of interest.

## Publisher’s note

All claims expressed in this article are solely those of the authors and do not necessarily represent those of their affiliated organizations, or those of the publisher, the editors and the reviewers. Any product that may be evaluated in this article, or claim that may be made by its manufacturer, is not guaranteed or endorsed by the publisher.

## Abbreviations

CRC: colorectal cancer
SPSS: Statistical package for social science
SD: Standard deviation
SE: Standard error
CI: Confidence interval
FDRs: first-degree relatives
